# Culturally appropriate consent processes for community-driven indigenous child health research: a scoping review

**DOI:** 10.1186/s12910-023-00996-9

**Published:** 2024-01-03

**Authors:** Cindy Peltier, Sarah Dickson, Viviane Grandpierre, Irina Oltean, Lorrilee McGregor, Emilie Hageltorn, Nancy L. Young

**Affiliations:** 1 Schulich School of Education, Nipissing University, North Bay, ON Canada; 2 Children’s Hospital of Eastern Ontario (CHEO) Research Institute, Ottawa, ON Canada; 3 Department of Health Research Methods, Evidence and Impact, McMaster University, Hamilton, ON Canada; 4 Northern Ontario School of Medicine, Sudbury, ON Canada; 5 Faculty of Chemistry, University of New Brunswick, Fredericton, NB Canada; 6 Faculty of Medicine, University of Ottawa, Ottawa, ON Canada

**Keywords:** Indigenous peoples, Minors, Parental consent, Ethics, research, Child, Adolescent, Health disparate, minority and vulnerable populations

## Abstract

**Background:**

Current requirements for ethical research in Canada, specifically the standard of active or signed parental consent, can leave Indigenous children and youth with inequitable access to research opportunities or health screening. Our objective was to examine the literature to identify culturally safe research consent processes that respect the rights of Indigenous children, the rights and responsibilities of parents or caregivers, and community protocols.

**Methods:**

We followed PRISMA guidelines and Arksey and O’Malley’s approach for charting and synthesizing evidence. We searched MEDLINE, PsycINFO, ERIC, CINAHL, Google Scholar, Web of Science, Informit Indigenous Collection, Bibliography of Native North Americans, and Sociological Abstracts. We included peer-reviewed primary and theoretical research articles written in English from January 1, 2000, to March 31, 2022, examining Indigenous approaches for obtaining informed consent from parents, families, children, or youth. Eligible records were uploaded to Covidence for title and abstract screening. We appraised the findings using a Two-Eyed Seeing approach. These findings were inductively coded using NVivo 12 and analyzed thematically.

**Results:**

We identified 2,984 records and 11 eligible studies were included after screening. Three key recommendations emerged: *addressing tensions in the ethics of consent*, *embracing wise practices*, and *using relational approaches to consent*. *Tensions in consent* concerned Research Ethics Board consent requirements that fall short of protecting Indigenous children and communities when culturally incongruent. *Wise practices* included allowing parents and children to consent together, land-based consenting, and involving communities in decision-making. Using *relational approaches to consent* embodied community engagement and relationship building while acknowledging consent for Indigenous children cannot be obtained in isolation from family and community.

**Conclusions:**

Very few studies discussed obtaining child consent in Indigenous communities. While Indigenous communities are not a monolith, the literature identified a need for community-driven, decolonized consent processes prioritizing Indigenous values and protocols. Further research is needed to examine nuances of Indigenized consent processes and determine how to operationalize them, enabling culturally appropriate, equitable access to research and services for all Indigenous children.

## Background

### Locating the research team

Among many Indigenous nations, introductions are customary to establish relationships. Self-location is a necessary and respectful means of promoting accountability, authenticity, and connection with Indigenous Peoples and lands. CP is Anishinaabe from Wiikwemkoong Unceded Territory and Nipissing First Nation. LM is an Anishinaabe from Whitefish River First Nation. SD is a Canadian woman with no Indigenous heritage. VG identifies as a first-generation Hungarian Canadian, IO self-identifies as Romanian Canadian, EH is Canadian, and NY has no Indigenous heritage and is on her journey to become an ally.

### Rationale

Our scoping review aimed to identify peer-reviewed literature about Indigenous consent practices in health research that supports children’s right to participate in research while respecting caregiver rights and community protocols. The impetus for this review was challenges experienced in obtaining the required active parental consent for vulnerable children and youth to access a mental wellness assessment and necessary care. Our research team members have engaged in community-based, collaborative research with and for Indigenous children for over a decade, leading to the co-creation of a novel wellness assessment, the *Aanish Naa Gegii – the Children’s Health and Well-being Measure* (ACHWM) [1], now welcomed by more than 50 communities. The ACHWM is a tablet-based, accessible screening tool that provides an overview of a child’s self-reported spiritual, emotional, physical and mental health. Once completed, a real-time report of results offers a culturally appropriate, visual snapshot of child wellness and promotes timely access to resources, including health workers, health promotion programs, natural helpers, and clinical support [2].

Health workers in many communities implementing the ACHWM must obtain caregiver consent for children under twelve to implement the measure and its follow-up. However, requirements like active parental consent can leave children and youth with inequitable access to research opportunities [3] or health screening like the ACHWM. Indigenous peoples are already underrepresented in health research [4, 5], further complicated by the issue of consent for children. Few legal statutes outline a specific age of consent; for example, Ontario’s Education Act requires parental consent for IQ and personality testing of students under eighteen [6]. Consent process improvements can help increase Indigenous Peoples’ representation in research [4, 5].

Western concepts like decision-making capacity and parental or authorized third-party signed consent [7] are expectations of ethical research outlined in many institutional and national research policies, including the Tri-Council Policy Statement Second Edition (TCPS-2) [8]. TCPS-2 defaults to parental consent and supersedes Indigenous notions of collective decision-making for research participation [3, 7, 9]. While guiding principles for Indigenous research (e.g., respect for community customs, community engagement, and consultation with Elders and knowledge holders) have made their way into the TCPS-2, examination of consent processes for Indigenous children has not yet been addressed by these guidelines. Baydala et al. [7] highlight the familial, community and cultural protective contexts that embed children and consent. The authors share obtaining child assent in isolation is problematic as it disregards collective and relational decision-making in Indigenous communities and can promote culturally unsafe situations [7]. This scoping review explored whether specific Indigenous child consent processes exist and how best to engage with communities regarding consent.

### Objectives

The scoping review objectives were to (1) investigate consent practices among Indigenous children and families and (2) summarize any key recommendations to guide future research. Our scoping review builds on previous work examining seeking research consent with Indigenous communities and responds to a call for research seeking consent with children [10] for health research.

## Methods

The research question guiding our scoping review was: What are culturally safe consent processes that respect the rights of Indigenous children, community protocols, and the rights and responsibilities of parents in the context of health research? We followed the PRISMA Scoping Review (PRISMA-ScR) guidelines [11] and Arksey and O’Malley’s approach [12] for charting and synthesizing data from articles into codes, subthemes, and themes to report meaningful and purpose-oriented results. Our protocol [13] is available at Open Science Framework Preprints.

**Fig. 1:**
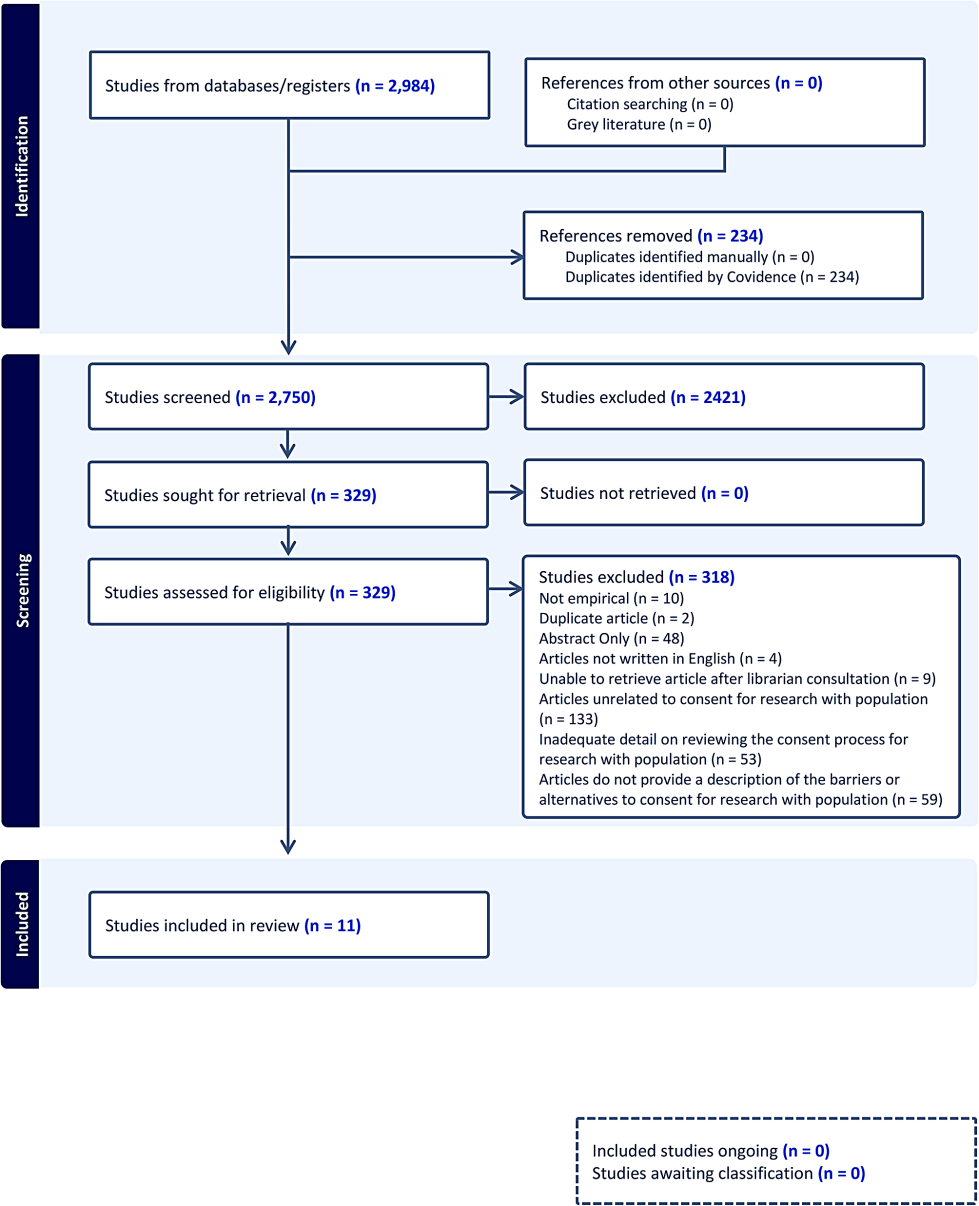
PRISMA diagram

We consulted librarians to identify relevant databases and effective search strategies, and included peer-reviewed primary and theoretical research in English from January 1, 2000, to March 31, 2022, examining Indigenous approaches for obtaining informed consent from parents, families, children, or youth. We included records that: (1) described detailed consent for research with Indigenous children, youth, parents, or families; (2) evaluated consent (e.g., barriers) for research; or (3) explained protocols for obtaining consent in health research (Fig. 1). We included literature from 2000 to 2021 to reflect the growth in foundational Indigenous research texts. Our search did not filter by geography. Consent had to be stated explicitly in titles or abstracts. Indigenous communities typically consider youth to extend up to the age of thirty, and thus we chose to focus on those under thirty. If the ethnic backgrounds of participants were ambiguous, we consulted the Native Land Digital Map [14] to determine if the population could be Indigenous.

We applied the search string: [consent* OR assent*] AND [Indigenous OR Aboriginal* OR “First Nation*” OR Metis OR Inuit* OR Indian* OR Native*] AND [famil* OR child* OR youth* OR adolescen* OR parent*] to available bibliographic databases including Medical Literature Analysis and Retrieval System Online (MEDLINE), Psychological Information Database (PsycINFO), Educational Resources Information Centre (ERIC), the Cumulative Index for Nursing and Allied Health Literature (CINAHL), Web of Science, Informit Indigenous Collection (IIC), Bibliography of Native North Americans, and Sociology Abstracts. Applying the search string in Google Scholar, we limited to the first 100 records. We excluded duplicate articles, those not written in English, that did not include a clear description of consent process and/or barriers or alternatives to consent, were limited to an abstract, were not based in evidence, or could not be accessed. We uploaded eligible papers to Covidence [15]. CP, IO, and EH performed Level I (title and abstract) screening. Level II (full text) screening was performed in three separate article batches by independent reviewers CP, IO, and VG.

The remaining papers were appraised using an approach that was informed by Elder Albert Marshall’s concept of Two-Eyed Seeing [16] combining the elements of Western and Indigenous tools to promote appraisal that is culturally relevant. We combined elements of the Critical Appraisal Skills Programme (CASP) checklists [17] with an adaptation of the Aboriginal and Torres Strait Islander Quality Appraisal Tool (ATSI-QAT) [18] to appraise the articles. The CASP Checklist is a 10-item tool designed to assess the quality of qualitative research from a Western perspective [17]. The 14-item ATSI-QAT was developed to evaluate quality from an Indigenous perspective and addressed a lack of appraisal tools relevant to Indigenous peoples and research ethics [18]. Our adaption of the ATSI-QAT reflects an Anishinaabek understanding. CP, VG, and IO piloted the modified tool on three studies, reviewed in triplicate by CP, VG, and IO. We will share the development and results in a separate publication.

## Results

### Selection of evidence sources

Our research team members screened 2,984 records, removing 234 duplicates. 2,750 articles proceeded to Level 1 screening performed independently by three researchers (CP, IO and EH). 329 articles met the inclusion criteria and moved to Level 2 review, where 318 articles were excluded for lack of relevance (n = 133), lack of description of barriers or alternatives to consent (n = 59), insufficient detail on the consent process (n = 53), abstract only (n = 48), not empirical (n = 10), unretrievable after librarian consultation (n = 9), not written in English (n = 4) or duplication (n = 2). Eleven articles remained in the final review (Fig. 1).

### Analysis of articles

Table 1 summarizes the characteristics of the eleven studies. These studies were published between 2005 and 2021 in Canada (n = 4), the United States (n = 4), Australia (n = 2), and New Zealand (n = 1). Eight interventional studies documented challenges and wise consent practices. Three studies involved focus groups and discussed ethical considerations and tensions inherent in conducting child or youth research with Indigenous communities. No studies reported exclusively on children; three had child or youth populations. Three studies sampled adult researchers or teachers. One study included a waiver for parental consent, while the majority relied on conventional informed consent. Refer to Table 2 for relevant study findings.

**Table 1:** Study Characteristics

Author	Year	Location	Objective	Study Population	# of Children/ Youth	# of Adults	Child/Youth Consent Method	Parental Consent Method
Stiffman et al.	2005	USA	To report on the pressures between research and ethics in a study of service use in AI^a^ youth.	Youth, 12–19	401	0	Written consent	Written
Panagiotopoulos et al.	2007	Canada	To implement a children’s diabetes screening program.	Children, 6–18	29	0	Written assent	Written
Baydala et al.	2011	Canada	To discuss the challenges of gathering child assent during a CBPR^b^ project.	Research team members	0	7	N/A	N/A
Rose et al.	2011	New Zealand	To gather ideas on promoting return of consent forms and vaccine uptake among minority groups.	School staff	0	456	N/A	N/A
Fletcher et al.	2012	Canada	To develop recommendations for managing ethical consent in research with Aboriginal communities.	Research team members	0	7	N/A	N/A
Jardine & James	2012	Canada	To examine benefits, limitations and ethical issues when conducting peer participatory research.	Students, Grades 2–12	48	0	Oral assent	Written/ oral
Yao et al.	2018	USA	To test a text message intervention to promote sexual health among AI^a^ and Alaska Native youth.	Youth, 15–18	408	0	Texted consent	Waived
Chadwick et al.	2019	USA	To test financial incentives for physical activity by overweight/obese adolescents.	AI^a^ youth, 11–20	116	0	Consent	Written
Wagner et al.	2020	Australia	To assess a cognitive regulation intervention in remote Australian Aboriginal schools.	Students, Grades 1–6	271	440	Written consent	Written
Anderson et al.	2021	Australia	To evaluate the effectiveness and feasibility of online yarning circles with ATSI^c^ Australian youth.	ATSI^c^ youth, 18–24	21	0	Email or verbal consent	N/A
Siller et al.	2021	USA	To explore the reactions of Native American adolescents to research on sexual violence.	Students, Grades 6–12	149	0	Written assent	Written

^a^American Indian; ^b^Community-based participatory research; ^c^Aboriginal and Torres Strait Islander

**Table 2:** Relevant Child/Youth Consent Study Findings

Author	Challenges and barriers to informed consent	Considerations for obtaining consent	Key Findings/Wise Practices
Stiffman et al.	- Some providers required parental permission to offer services, even if youth were in danger. - The consent form was not deemed suitable despite community consultation.	- Families could consent via signing and mailing a pre-stamped postcard or in-person pre-interview.	- Interviewers used home visits to explain the study, obtain parent/guardian and youth consent, and conduct the interview.
Panagiotopoulos et al.	- Child welfare is the Elder’s responsibility, and obtaining informed assent directly from children was foreign. - The authors noted many sociological, ethical, and practical challenges.	- Community members required sufficient information (letters, preliminary clinic visits) before participating. - Confidence/trust were key facilitators. - Pre-intervention trips ensured “appropriate dialogue, care, respect and planning"^3^.	- Timing must be flexible and on the community’s terms. - Multi-lateral support is required (Elders, hereditary and elected Chiefs, band council, school, and health leaders). - Senior investigators met with community Elders and hosted a joint community feast.
Baydala et al.	- Differences in prioritizing individual vs. collective rights. - “Individualistic” research values disrupted traditional roles. - Children’s assent was considered unnecessary after obtaining community and parental consent. - Children were placed “in conflict with some of their kinship responsibilities”^1^ when asked to challenge parents’ decisions. - “Trust remains an issue when using written forms of agreement with Western institutions.”^1^	- Community researchers advised that home visits were ideal to obtain parental consent. - Visits were carried out by researchers who, in most cases, were also family members. - Children’s right to refuse was essential.	- Child assent should be conducted in culturally appropriate settings and reflect community values.
Rose et al.	- The consent rate was lower for students with lower socioeconomic backgrounds.	- The burden of obtaining parental consent should not be placed on students. - Tech (website, texting) and incentives (voucher, badge, prize draw) can be used to encourage the return of consent forms. - Home visits can be used for families who have not returned their consent forms.	- Information was provided through face-to-face meetings and in local languages.
Fletcher et al.	- Western REB processes presented logistical and process challenges. Insufficient money and time were allocated to consent processes. - Wording, document length and use of medical/ research terms in consent forms were all challenges. The term “risk” brought up past research injustices. - Written consent caused discomfort, and relationship-based oral consent was preferred. - “The current process is not meeting the stated objectives of safety and informed consent as typically envisioned by REBs.“^5^	- Local researchers recommended respectful and informational home visits by a community-based team member. - Information sheets should be left with parents. - Involving community members in the research process fostered partnership and trust. - Consenting hinged on multiple home visits lasting 1–2 h each. - Issues of guardianship and family structure had to be considered sensitively.	- After observing that written consent made parents and community researchers uncomfortable, oral consent was recommended. Ideally, this would be land-based. - Consenting should “reinforce culturally based ethical norms and consent practices rather than negotiated as an add-on to academic institutional practices.”^5^ - Early involvement of Elders and community leaders fostered spiritual, political, and psychological protection. - Relational consent processes reaffirmed kinship with community researchers, including offering tobacco to leaders and Elders.
Jardine & James	- Written consent contradicts Aboriginal approaches to research in many Northern communities. - Determining who should consent on behalf of minors is challenging in communities where guardianship may not be formally recognized.	- Although parental/guardian permission was obtained, researchers obtained oral assent from students.	- Youth/student researchers were trained to obtain consent and interview other students.
Yao et al.	- Community members raised privacy and confidentiality concerns.	- A waiver allowed teens aged 15–18 to participate without parental consent. - Teens who had already subscribed to the study’s texting service were invited/consented via text.	- Text messaging was used to reach teens effectively.
Chadwick et al.	NR	- Using a video allowed consent to be standardized across multiple research sites.	- Community leaders identified culturally appropriate ways to facilitate the trial’s implementation. - The tribal REB was designated the board of record.
Wagner et al.	- Since recruitment was school-based, vulnerable children could be denied participation opportunities due to school absences. - Low school attendance was due to funerals, medical appointments, and cultural events.	- Community researchers verbally translated study materials into appropriate languages, as required.	- Research assistants and community researchers conducted joint home visits to seek parental consent.
Anderson et al.	- Ensuring robust processes for informed consent, withdrawal and debriefing in an online environment	- Consent via email or audio-recorded verbal consent at Online Yarning Circle	NR
Siller et al.	NR	- Researchers incentivized returning consent forms regardless of consent or participation.	- Multiple ways to return consent forms (email, text or in-person)

NR = Not reported

### Thematic findings

Due to an absence of literature focusing on Indigenous consenting practices in child, youth and caregiver populations, our team analyzed the findings through iterative, inductive coding rather than with any pre-determined framework. We identified prevalent themes by searching for patterns of significance and relationships between coding categories. Using Nvivo 12 [19], SD performed the initial coding, and CP reviewed the codes. Analysis was performed by CP and approved by all authors. Initial coding resulted in 110 unique codes organized into three themes: *Wise Practices for Consent*, *Tensions in the Ethics of Consent*, and *Relational Approaches to Consent*.

### Wise practices^1^ for consent

This theme embodied the sharing of wise consent practices and encompassed 39 codes and four subthemes: ‘language’, ‘accommodations in consent/assent process’, ‘parent/caregiver considerations’, and ‘child/youth considerations’.

The ‘language’ subtheme concerned accommodations for literacy and translation in the consent process. Two papers [9, 20] highlighted using plain language for written and oral communication. Three studies [9, 21, 22] discussed translation respecting Indigenous languages and dialects for meaningful consent, where hiring community-based researchers with skills in translation and knowledge of cultural protocols was vital [22].

‘Accommodations in consent/assent process’ as a subtheme concerned flexibility in setting, process, and time. Several authors recommended adapting consent practices by consulting Indigenous communities [7, 9, 20, 21, 23, 24]. While accommodations might diverge from current Western Research Ethics Board (REB) standards, they align well with tenets of community-based participatory research (CBPR) and Indigenous paradigms [9]. Less intimidating consent settings such as group, home visits, or land-based gatherings allowed participants to engage on their terms and to develop relationships before consenting. Home visits led by community-based researchers required flexibility in funding and time allocated for consent involving multiple interactions with parents and families [7, 9, 20–22]. Fletcher et al. [9] and Panagiotopoulos et al. [24] recommended leaving consenting materials with parents to process for at least 24 hours. Researchers suggested a variety of strategies for obtaining and returning consent forms. In Stiffman et al. [20], parents and children signed the same pre-stamped postcard and returned it via mail, allowing time to consider and discuss before jointly consenting. In the past, signing research documents has harmed Indigenous peoples; thus, flexibility in oral or written consent choices is significant [7, 9, 20] with a preference for relationship-based, verbal consent [9, 25].

The ‘parent/caregiver considerations’ subtheme focused on family living situations and age of consent. Identifying appropriate consent providers was challenging when children lived separately from their parents or in foster-care settings [9, 25], especially when children who can benefit from interventions live in situations where consenting is complex. Age and waiving caregiver consent were issues for youth research participation. In the study by Yao et al. [26], the sensitive nature of a sexual health intervention and privacy and confidentiality concerns, parental consent was waived for youth 15 to 18 years.

The role of schools was prominent in the ‘child/youth consent considerations’ subtheme. Seven studies mentioned school involvement as an intervention location or place of consent; however, school involvement may be inappropriate for some studies. Rose et al. [21] found lines blurred between school requirements and voluntary human papillomavirus research, and consent rates decreased in lower-income families, highlighting inequity. Wagner et al. [22] speculated that absenteeism prevented vulnerable children from participating in a cognitive intervention. James and Jardine [25] found reliability increased in a less intimidating environment of a youth-led study of peer perceptions of smoking. Sending consent materials home might be an opportunity to facilitate consent conversations, but these authors caution against overburdening the vulnerable and exacerbating inequity.

### Tensions in the ethics of consent

This second theme encompassed 30 codes and four subthemes, including ‘ethics review,’ ‘technology-related consent,’ ‘challenges to the consent process,’ and ‘ethical tensions.’

The ‘ethics review’ subtheme concerned the choice of REB review, where researchers advocated for balancing REB requirements with community and cultural understandings of ethics, highlighting Indigenous self-determining consent processes. Only [9] opted for an ethics review via university REB. More typical was co-review by university and tribal REBs, with the university REB as the board of record. Chadwick et al. [23] elected a tribal REB as the board of record, meaning the community presided over study protocol and ethical conduct. This designation was essential since all study activities occurred within the Nation, and participants were patients of the Nation’s Health Service.

For the subtheme ‘technology-related consent,’ researchers intended to increase recruitment, consenting, and participant comfort with technology. Text messages reminded parents and students to return consent forms [21, 27] and allowed parents/youth to return forms via email or text [28]. Yao et al. [26] used texting to recruit older youth who signed up to receive sexual health program messages. Anderson et al. [27] conducted online yarning circles with youth who reported increased comfort in declining uncomfortable questions. These authors discussed ethical concerns with using technology for child and youth research, including rigorous consenting and withdrawal processes, privacy, and confidentiality, accommodating participant comfort with video sharing, and data security [27].

The ‘challenges to the consent process’ subtheme involved defaulting to written consent, including complex wording, excessive length and details in forms requiring several follow-ups before consenting [7, 9]. Using the term risk in consent forms had negative connotations, and these forms failed to convey community-level risks [7, 9]. Community researchers in Fletcher et al. [9] were uncomfortable with the consent process, noting Western REB guidelines failed to ensure the safety of participants despite believing the research itself was ethical and beneficial. Similarly, service providers in Stiffman et al. [20] found the consent form inadequate despite being consulted, suggesting cultural incongruence with written consent rather than specific content.

The ‘ethical tensions’ subtheme focused on research prioritizing Western values of autonomy, individualism, and caregiver consent. In contrast, Indigenous peoples value collective decision-making according to kinship systems, community, and elder involvement, with decisions rooted in their impact on the community [7, 9]. According to Baydala et al. [7], when obtaining their assent, children questioned whether their parents or teachers had already consented. Community researchers attributed this to a child’s understanding of community consent protocols, arguing that the assent process threatened cultural safety [7]. The values underpinning consent processes should determine decision-making for and with children [7]. Fletcher et al. [9] noted that the extent of child autonomy will depend on the nature of the research. Still, child consent should emphasize a relational approach [9].

### Relational approaches to consent

This theme comprised 39 codes and seven subthemes addressed within two considerations: (1) relationships in consent inclusive of the subthemes: ‘community-based research team members’, ‘researcher concerns or issues’, ‘connections in all relationships’, ‘community engagement’, and ‘relationship building’, and (2) methodologies for relational consent involving the subthemes: ‘CBPR’ and ‘decolonized/Indigenist research practices’.

#### Relationships in consent

The subtheme ‘community-based research team members’ reflected valuing their involvement in consenting for many reasons, including building trust [7, 9, 22], ensuring consistent communication [7, 23, 24], diminishing power influences [25, 27], facilitating relationships with parents and communities [22], developing research capacity [22], and providing knowledge of community protocols or language translation [9, 22]. An expectation was that community-based research team members fostered deeper connections with parents, children, and the community as familiar and trusted personnel.

In the subtheme, ‘researcher concerns or issues,’ researchers expressed feeling conflicted when requesting signatures from family or community members. They felt uneasy about some wording in consent forms and rightfully asserted community control over cultural knowledge [7]. Relationships brokered between Indigenous and non-Indigenous research team members and research participants were essential for cultural safety [22] and required investments of time, training, and ongoing support [7, 9, 22]. Two studies recommended training for REBs in Indigenous child consent or assent [7, 9]. Researcher reflexivity and positionality were essential to outlining perspectives and strengths [27].

In the ‘connections in all relationships’ subtheme, kinship systems underscored the relationships between community-based researchers, children, caregivers, Elders, families, and clans [7, 9, 24]. In Baydala et al. [7], one community-based researcher objected to researcher bias, arguing strong kinship ties were beneficial to obtaining consent or assent. Moreover, Fletcher et al. [9] highlighted the importance of collective decision-making and Elder or community leader approval was deemed a form of protection when participating in research.

The ‘community engagement’ subtheme encompassed approvals from Elders and community leaders [7, 9, 20, 22–24] and formal endorsements such as Band Council Resolutions [7]. Baydala et al. [7] encouraged collectively obtaining consent from the community and caregivers and assent from children in land-based activities. While Stiffman et al. [20] implemented a research implementation team to ensure ongoing partnership, others advocated for community engagement and input more generally in all stages of research [7, 28]. There were concerns about confidentiality in smaller communities [20], especially with sensitive issues. Fletcher et al. [9] stressed the importance of diversity in consent protocols such as offering tobacco, oral consent, group consent, or consensus grounded in respect, reciprocity, and cultural teachings. Informed consent should be rooted in community cultural protocols rather than as a supplement to Western processes [9]. Self-determination, community ownership and informed consent/assent process control were encouraged [7, 9].

‘Relationship building’ as a subtheme concerned relationships between Indigenous and non-Indigenous team members [9, 22], between researchers and Indigenous communities [9, 22, 24, 28], and relationships formed during informed consent processes [9]. In some studies, research team members’ community connections were assets for the consent process [24, 26, 27]. In others, it was necessary to acknowledge relationship-building as a strength instead of coercion [7, 9].

#### Methodologies for relational consent

CBPR principles shared in the studies reviewed included community involvement in planning [7, 23, 24, 28], community collaboration [23, 27, 28], shared decision-making [7, 20, 23, 25, 28], community involvement in analysis of results and providing ongoing teaching [9] and relating research to community-identified needs [28]. A benefit of CBPR is two-way, bi-directional or co-learning between researchers and community members [9, 22]. Separate but related concepts were capacity strengthening in research processes [22, 25, 27], capacity development in research ethics [20, 23], promoting community understanding of screening results [24], and cross-cultural learning for non-Indigenous researchers [9]. The involvement of community members in research builds trust and depends on relational connections [7, 9] or pre-existing relationships between researchers and the community [24, 26]. There must be a willingness to listen, ongoing mutual learning, and shared ownership over all aspects of research, including consent processes [7, 9, 24].

In the reviewed studies, decolonized/Indigenist research practices included offering tobacco as part of the protocol for consent with Elders [9], conducting research discussions and activities in an ethical space [9], and employing culturally appropriate methods such as Yarning [27], sharing circles [7], and focus groups [9]. Being on the land was proposed as a relational, safe space for discussions about caregiver consent and child assent [7, 9]. Following cultural protocols alongside standard research protocols were essential to the success of the reviewed studies [7, 9, 22–24, 26, 27]. For studies employing decolonized/Indigenist research practices, cultural safety was both a research aim, and a desired outcome for consenting with children, youth, caregivers, and communities [7, 9, 22, 27].

## Discussion

We initiated this review in response to communities’ observations that some of the most vulnerable children and youth were experiencing inequitable access to strengths-based research opportunities due to an overreliance on a written parental consent process. The review and analysis of findings reflect a response to our research question while respecting new conceptualizations, challenges, strategies, or practices informing consent in Indigenous contexts globally. We synthesized the results in three broad themes. A finding worth emphasizing is that all but one study depended on conventional informed consent, confirming our initial observation that relying solely on written permission from parents is problematic and limits Indigenous understandings of consent. A single youth study [26] reported innovative consent via waived parental consent. No study reported exclusively on child consent, signaling a gap in the literature.

This review aimed to identify wise practices for researchers. Four studies [7, 21, 22], and [20] advocated for meaningful oral and written communication in consent by using plain English and respecting Indigenous languages by offering translations when requested. To support the reclamation of Indigenous languages, we would add a caveat for including local languages in research processes as a respectful and reciprocal practice. Linda Tuhiwai Smith’s [29] often-quoted sentiment, for Indigenous Peoples, research is a dirty word, is now a universal message that research, and we argue, consent processes continue to alienate and create discomfort. Diminishing the stigma around research while enabling Indigenous peoples’ engagement on their terms requires a commitment to trust-building and offering culturally appropriate consent processes.

Consent is often the first encounter with research participants. Appropriate settings help to create safe spaces for consenting and are conducive to positive interactions. For youth, the environment could be virtual. Anderson reports interacting online increased psychological safety and comfort for youth participants. While technology is a promising avenue for improving recruitment and comfort, it should not compromise ethics. For instance, virtual accommodation might allow participants to turn cameras off during research interactions, respecting privacy. Travel to locations preferred by participants can build trust and foster good relationships but requires investments of the researcher and the project budget. Research should support relational connections, offering families and communities land-based opportunities grounded in cultural wisdom. Accommodation is possible with planning and reciprocity in mind.

Historical legacies continue to have implications for safe consenting processes. The rights of Indigenous parents and communities to raise and educate their children was historically denied through racist, colonialist policies. Residential schools [7] forcibly removed Indigenous children from their homes and placed them in church-run institutions. During the Sixties Scoop, children were removed from their homes and became wards of the state. A respectful consent process should enable discussions and decision-making with relatives or occur in kinship circles instead of relying on one-on-one interactions with strangers [7]. Moreover, signing documents has been problematic and created ongoing mistrust. It is critical to offer multiple consent modes, including oral or written consent with numerous options for returning forms, technology-based, cultural protocols like passing tobacco, and enabling families or communities to consent together. Researchers should acknowledge that Elders and community leaders deem what is acceptable in communities, and their consent supersedes all others. Theoretically, Western and Indigenous worldviews emphasize upholding children’s rights and safety in research; however, individualistic notions of rights can place Indigenous children in culturally incongruent situations. Consent processes should reflect community values, honouring caregivers’, elders’, and community leaders’ guidance, wisdom, and protection.

A balance in Western and Indigenous ways of knowing is essential to conduct research and consent ‘*in a good way.’* Our review demonstrates that current practices prioritize Western values [9]. While many studies consulted communities and Elders, this was often a prelude to formal consent processes that researchers minimally adapted for Indigenous peoples. Indigenous protocols were secondary to Western REB procedures. In Fletcher et al. [9], community researchers voiced concerns about asking parents to conform to processes that made them uncomfortable. Community-based researchers helped mitigate the discomfort and mistrust involved with written consent, but this came at the cost of cultural safety. Righting the imbalance in ways of knowing can avoid perpetuating further harm [9].

### Strengths, limitations and key implications

This scoping review addresses a gap in the literature regarding wise practices for seeking consent from Indigenous children, families, and communities. An implication of this review is highlighting the chasm in health research between understandings of institutional and community research ethics as they pertain to child, youth, and community health. While institutional REBs focus on individual consent and rights, our study sheds light on collective, whole-community decision-making desired by Indigenous communities seeking self-determining processes. Still, the apparent disregard for culturally relevant consent practices in the reviewed studies indicates a knowledge gap. Future studies should examine non-academic grey literature in detail, as valuable research conducted by Indigenous communities and organizations may not appear in peer-reviewed literature.

### Future directions

Our next step will report the results of the quality appraisal component, honouring Two-Eyed Seeing by examining quality appraisals from Indigenous (ATSI-QAT) [18] and Western (CASP) [17] perspectives. A follow-up inquiry will interview key informants to determine how consent is given and refused in Indigenous societies to enhance implementation and access to the ACHWM.

## Conclusions

From the limited studies remaining in our scoping review, we share three recommendations: (1) Listen to the guidance of Indigenous peoples and follow wise practices for culturally relevant consent, (2) Create action needed to address the tensions and chasm between community and institutional research ethics, and (3) follow a relational approach to consent that involves wider kinship networks inclusive of children, families, and communities. A deeper understanding of Indigenous consent is required. Further research will contribute to scholarship about consent and will have implications for reclaiming Indigenous governance processes about research practice. We see addressing this gap in Indigenous consent knowledge as meaningful support for United Nations Declaration on the Rights of Indigenous Peoples (UNDRIP) Article 34 [30],

“Indigenous peoples have the right to promote, develop and maintain their institutional structures and their distinctive customs, spirituality, traditions, procedures, practices and, in the cases where they exist, juridical systems or customs, in accordance with international human rights standards.”

## Data Availability

All data generated or analyzed during this study are included in this published article.

## Abbreviations

ACHWM: Aaniish Naa Gegii: the Children’s Health and Well-being Measure
TCPS2: Tri-Council Policy Statement Second Edition
PRISMA: Preferred Reporting Items for Systematic Reviews and Meta-Analyses
MEDLINE: Medical Literature Analysis and Retrieval System Online
PsycINFO: Psychological Information Database
ERIC: Educational Resources Information Centre
CINAHL: Cumulative Index for Nursing and Allied Health Literature
IIC: Informit Indigenous Collection
CASP: Critical Appraisal Skills Programme
ATSI-QAT: Aboriginal and Torres Strait Islander Quality Appraisal Tool
CBPR: Community-Based Participatory Research
REB: Research Ethics Board
UNDRIP: United Nations Declaration on the Rights of Indigenous Peoples

## Footnotes

1 The decision to frame recommendations as ‘wise’- rather than ‘best’ practices offers space for variability and diversity in Indigenous communities and contexts. Rather than being prescriptive, the phrase wise practices implies that not all practices will be the best for all but instead may be situation- or community- dependent.
